# Potential pharmacological effect of Quercetin Phytosome™ in the management of hyperuricemia: results from real-life clinical studies

**DOI:** 10.3389/fnut.2025.1519459

**Published:** 2025-02-07

**Authors:** Francesco Di Pierro, Fazle Rabbani, Meherullah Tareen, Roohi Nigar, Amjad Khan, Nicola Zerbinati, Maria L. Tanda, Massimiliano Cazzaniga, Alexander Bertuccioli, Paolo Falasca, Gabriele Damiani, Nicola Villanova

**Affiliations:** ^1^Microbiota International Clinical Society, Torino, Italy; ^2^Department of Scientific and Research, Velleja Research, Milano, Italy; ^3^Department of Medicine and Technological Innovation, University of Insubria, Varese, Italy; ^4^Department of Psychiatry, Lady Reading Hospital, Peshawar, Pakistan; ^5^Department of Oncology, Bolan Medical College Hospital, Quetta, Pakistan; ^6^Department of Obstetrics and Gynecology, Bilawal Medical College, Jamshoro, Pakistan; ^7^Department of Oncology, University of Oxford, Oxford, United Kingdom; ^8^Endocrine Unit, Department of Medicine and Surgery, University of Insubria, Varese, Italy; ^9^Department of Biomolecular Sciences, University of Urbino Carlo Bo, Urbino, Italy; ^10^UOC Medicina Interna, Polo H1, ASL Rome, Italy; ^11^IRCCS-Azienda Ospedaliera di Bologna Sant' Orsola-Malpighi, Bologna, Italy

**Keywords:** hyperuricemia, cardiovascular disease, Quercetin Phytosome™, Quevir^®^, quercetin, metabolic health

## Abstract

**Background:**

Hyperuricemia is associated with several metabolic and cardiovascular disorders, and traditional treatments, such as xanthine oxidase (XO) inhibitors, often have limitations, such as severe hypersensitivity reactions or ineffectiveness in achieving target serum urate levels in some patients. Quercetin, a naturally occurring flavonoid, has shown potential as a hypouricemic agent through XO inhibition.

**Objective:**

This study aims to evaluate the potential hypouricemic effect of Quercetin Phytosome™ (QP) supplementation across three cohort studies involving healthy adults with various metabolic health profiles, exploring its potential as a safe, effective intervention for hyperuricemia.

**Methods:**

Clinical data collected in various clinics in Italy between September 2021 and April 2024 under real-life clinical settings from three distinct cohort studies, were analyzed. Cohort 1 consisted of 164 healthy participants (87 QP-treated, 77 probiotic *Streptococcus salivarius (S. salivarius)* K12-treated) who were monitored for 90 days. Cohort 2 included 22 mildly hyperuricemic adults with metabolic disorders receiving QP, while Cohort 3 comprised 64 obese adults with hypercholesterolemia, further divided into moderately hyperuricemic QP-treated group (*n* = 20), a moderately hyperuricemic Berberine Phytosome™ and monacolins (BM)-treated group (*n* = 22), and a normouricemic BM-treated group (*n* = 22). QP was administered at 400 mg of quercetin daily in all cohorts. Primary endpoints were reductions in serum uric acid levels, while secondary outcomes included effects on lipid profile, glycemia, liver enzymes, and treatment tolerability.

**Results:**

In Cohort 1, QP significantly reduced uric acid levels by 15.2% in males and 13.8% in females, with no significant changes observed in the probiotic group. Cohort 2 showed a significant 13.1% reduction in uric acid (*p* < 0.01) and a concurrent 10.2% reduction in triglycerides (*p* < 0.05). In Cohort 3, QP led to a 13.7% decrease in uric acid and a 20.8% reduction in triglycerides (*p* < 0.01), with no significant uric acid changes in the BM-treated group. QP was well tolerated across all cohorts, with minimal, transient side effects.

**Conclusion:**

QP supplementation demonstrates a significant hypouricemic effect. Additionally, triglyceride-lowering benefits were evident, particularly in metabolically compromised individuals (Cohorts 2 and 3), where these effects were statistically significant. With high tolerability, these findings highlight Quercetin Phytosome™'s potential as a safe adjunctive therapy for hyperuricemia management, meriting further investigation in larger, randomized trials to confirm its efficacy and safety.

**Clinical Trial Registration:**

clinicaltrials.gov, identifier NCT06652035.

## 1 Introduction

Hyperuricemia is a metabolic disorder characterized by elevated serum uric acid concentrations (1). Epidemiological studies and clinical trials have linked hyperuricemia to the development of various conditions, including chronic kidney disease, fatty liver disease, metabolic syndrome, hypertension, insulin resistance, obesity, type 2 diabetes (T2DM), cardiovascular and cerebrovascular disorders (2–4). However, Mendelian randomization studies have controversially shown no causal relationship between elevated uric acid levels and most of these conditions, with the exceptions of gout and kidney disease (5–10). Globally, hyperuricemia is typically defined as uric acid levels ≥7.0 mg/dL (416 μmol/L) in males and ≥ 6.0 mg/dL (357 μmol/L) in females, although the European Union defines it slightly lower: ≥6.8 mg/dL (404 μmol/L) for males and ≥5.7 mg/dL (339 μmol/L) for females (11, 12). Risk factors for hyperuricemia include: (i) dietary habits, such as alcohol consumption, high-purine diets, and fructose-rich intake; (ii) lifestyle and medical conditions, including obesity, insulin resistance, Down syndrome, kidney disease, hypertension, hypothyroidism, socioeconomic factors, and smoking history; and (iii) genetic predispositions and environmental factors (13–16).

From a physiological perspective, uric acid is the end product of purine nucleotide catabolism, with diverse biochemical roles. It acts both as an antioxidant and pro-oxidant, and has complex influences on aging, inflammation, and nitric oxide regulation (17). Current guidelines recommend maintaining long-term serum uric acid levels at or below 6.0 mg/dL (360 μmol/L) (18).

In cases of asymptomatic hyperuricemia, dietary interventions—particularly reducing alcohol, sugar-sweetened beverages, and high-purine foods like meat and seafood—can lower uric acid by 10–15% (19). However, complete purine restriction only reduces uric acid by about 1 mg/dL, making it an impractical sole strategy (1). Uric acid-lowering drugs can be divided into three main groups: (i) xanthine oxidase inhibitors (XO), which reduce uric acid synthesis; (ii) agents that promote uric acid excretion by inhibiting reabsorption; and (iii) drugs that regulate uric acid breakdown *via* exogenous uricase (20). Xanthine oxidase inhibitors, which decrease uric acid production from both endogenous and dietary purines, are the first-line treatment for hyperuricemia (21). The most commonly prescribed XO inhibitor, allopurinol, is often used with consideration of renal function. However, it can cause severe hypersensitivity reactions and skin conditions and is ineffective at reaching target serum urate levels in many patients (22). Newer non-purine XO inhibitors, such as febuxostat and topiroxostat, are increasingly used but are associated with side effects like muscle pain, gastrointestinal discomfort, skin rashes, diarrhea, polyarthritis, nasopharyngitis, and elevated liver enzymes (23, 24). Uricosuric agents, such as probenecid and benzbromarone, enhance uric acid excretion by inhibiting its reabsorption in the proximal tubule of the kidney. These drugs are second-line treatments, often used alongside XO inhibitors or for patients who cannot tolerate them (17). Uricase (urate oxidase) converts urate into allantoin, a more soluble compound, making it an attractive therapy for patients with gout that is difficult to manage with standard drugs. Though limited by factors like availability, cost, and immunogenicity, uricase shows promise as an enzyme replacement therapy for lowering the urate pool, potentially followed by maintenance with other urate-lowering treatments (25).

As a result of growing modern pharmacological evidence, and favorable safety profiles, there is increasing clinical interest in botanical pharmacological agents for managing hyperuricemia. Among these agents, quercetin—a well-studied flavonoid with diverse pharmacological properties—has shown promising results and demonstrated the ability to lower plasma uric acid levels (26). Research suggests that quercetin, and its metabolite quercetin-3′-sulfate, exhibit antihyperuricemic effect through the inhibition of XO (27, 28), the final step in the intracellular uric acid production. In a randomized, double-blind, placebo-controlled clinical trial, a daily intake of 544 mg of quercetin for 4-weeks significantly reduced uric acid levels in men, decreasing from 5.46 to 5.04 mg/dL (26). Despite these encouraging findings, clinical evidence remains limited regarding quercetin's effectiveness in managing hyperuricemia.

This study, based on analysis of retrospective clinical data from real-life clinics, evaluates the hypouricemic potential of oral supplemental quercetin, specifically Quercetin Phytosome™ (QP), in three distinct cohort studies involving healthy adults. QP is a highly bioavailable form of quercetin designed to overcome the poor solubility and limited absorption of standard formulations, which often restrict its clinical application (29). Additionally, QP has been investigated in several clinical trials for its broad pharmacological properties, including antioxidant, antiviral, immunomodulatory, and anti-inflammatory effects, further highlighting its therapeutic potential (30–33).

In cohort 1, participants received oral QP or the probiotic *Streptococcus salivarius* (*S. Salivarius*) K12 as immune booster supplement to enhance protection against COVID-19 infection/severe illness. Quercetin supports immune resilience through antioxidant and anti-inflammatory effects, modulating cytokine production and immune cell activity (34–36). *S. salivarius* modulates the oral microbiota, aiding immune function and reducing pro-inflammatory cytokines like IL-6, potentially mitigating excessive inflammation during SARS-CoV-2 infection (37–41).

In cohort 2, healthy adults received QP to improve metabolic health (42, 43). Quercetin helps manage hyperuricemia by inhibiting xanthine oxidase (27, 28), reducing uric acid production, and supports glycometabolic regulation by enhancing insulin sensitivity and modulating pathways like AMP-activated protein kinase (AMPK) and sirtuin-1 (SIRT1) (42–47). Additionally, it improves lipid profiles and mitigates inflammation through pathways like nuclear factor kappa B (NF-κB) and mitogen-activated protein kinase (MAPK) (42–47), addressing metabolic disorders such as obesity, dyslipidemia, and hypertension.

In cohort 3, participants with obesity and hypercholesterolemia received QP or a nutraceutical combination of Berberine Phytosome™ and monacolins (BM) for metabolic health management (48–51). Berberine activates AMPK to enhance fatty acid oxidation, reduce lipogenesis, and improve insulin sensitivity (48, 49, 51). It also modulates gut microbiota and improves lipid profiles by lowering total cholesterol, triglycerides, and LDL cholesterol while increasing HDL cholesterol (48, 51). Monacolin K, a natural HMG-CoA reductase inhibitor (50), reduces hepatic cholesterol synthesis, further lowering cholesterol and triglycerides. These complementary mechanisms provide a synergistic approach to addressing obesity and hypercholesterolemia (48–51).

The present study aims to enhance the understanding of QP's potential as a safe and effective pharmacological option for managing hyperuricemia. By evaluating its effects across varied clinical scenarios, this research highlights the versatility and therapeutic promise of QP in the management of hyperuricemia.

## 2 Materials and methods

To evaluate the potential hypouricemic effect of QP, retrospective clinical data were analyzed from three distinct cohorts of healthy adult participants who had received QP supplementation for health benefits under real-life conditions, rather than within the framework of a pre-designed study. The supplemental treatments were tailored to the therapeutic needs of the participants based on physician recommendations.

### 2.1 Cohort 1

The first cohort comprised a retrospective, cross-sectional, observational study of 164 participants who attended various clinics in Milan and Pesaro, Italy in real-life between September 2021 and December 2022, primarily aimed at enhancing protection against SARS-CoV-2 infection and/or severe COVID-19 illness (30–32, 40, 52). The participants were healthy individuals or simply overweight, aged between 18 and 65 years, with a BMI between 18.5 and 29.9 kg/m^2^, non-smokers, and consuming <3 units of alcohol per day. The participants received either oral supplemental QP (Quevir^®^ 500 mg tablet, manufactured by Pharmextracta S.p.A. Italy) (*n* = 87, 39 males and 48 females) or an orodispersible probiotic *S. salivarius* K12 (Bactoblis^®^ 1,000 mg tablet, manufactured by Pharmextracta S.p.A. Italy) (*n* = 77, 32 males and 45 females) tailored to their individual needs. Each Quevir^®^ 500 mg tablet contained 500 mg of QP, equivalent to 200 mg of pure bioavailable quercetin. Each Bactoblis^®^ 1,000 mg tablet contained at least 1 × 10^9^ CFU of *S. salivarius* K12 (ATCC BAA-1024). The recommended dose of the supplement was two tablets of QP per day (one every 12 h, total daily quercetin intake 400 mg) or one tablet daily of K12 (before bedtime) for 90 days. Regarding concurrent therapies, 28 participants (17.07%) (16 in the K12 group and 12 in the QP group) were on antihypertensive medications, while 22 (11 in each group) were on lipid-lowering drugs. Supplement was not advised to the participants if they had a diagnosis of endocrinological, metabolic, oncological, neurological, or inflammatory bowel diseases, gout, kidney stones, or plasma uric acid concentrations exceeding 7 mg/dL (hyperuricemia).

### 2.2 Cohort 2

The second cohort study was a prospective, single-group, cross-sectional observational analysis of 22 (13 males and 9 females) mildly hyperuricemic healthy adults (uric acid > 7 mg/dL) with concurrent metabolic disorders. Participants comorbidities included thyroid goiter (*n* = 4), prediabetes (*n* = 3), chronic asthenia (*n* = 1), pulmonary arterial hypertension (*n* = 2), T2DM (*n* = 7), obstructive sleep apnea syndrome (*n* = 1), hypothyroidism (*n* = 3), obesity (*n* = 8), metabolic syndrome (*n* = 4), hypertension (*n* = 11), and dyslipidemia (*n* = 6). These participants attended various clinics in Rome and Teramo in real-life between May 2023 and September 2023, and received QP (Quevir^®^ 500 mg tablet, same dosage as in cohort 1) as an add-on to their usual treatment, tailored to their individual needs, to improve their metabolic health. Participants were aged 18–75 years, and supplement was advised with no restrictions on BMI, alcohol consumption, or smoking habits. Those on uric acid-lowering drugs and the presence of oncological, neurological, or bowel inflammatory diseases, were excluded from supplemental therapy.

### 2.3 Cohort 3

The third cohort study was a prospective, observational study comprised of 64 healthy adults (38 males and 26 females), all diagnosed with obesity (for a minimum of 5 years) and hypercholesterolemia, and included both moderately hyperuricemic (average uric acid > 8 mg/dL) and normouricemic individuals (uric acid < 7 mg/dL). These participants attended the S. Orsola Hospital in Bologna and various clinics in Milan in real-life between October 2023 and April 2024, for treatment of their metabolic health. Amongst the 64 cases, 20 moderately hyperuricemic participants (10 males and 10 females, referred here as group 1) were treated with QP (Quevir^®^ 500 mg tablet, same dosage regimen as in cohorts 1 and 2). A second group consisting of 22 moderately hyperuricemic adults (uric acid > 8 mg/dL) (referred here as group 2) were treated with a nutraceutical containing Berberine Phytosome™ and monacolins (BM) (Berberol K^®^ 1.25 g tablet, manufactured by Pharmextracta S.p.A. Italy), as one tablet a day for 90 days, to improve their metabolic health. A third group comprised of 22 normouricemic adults (uric acid < 7 mg/dL) were also treated with BM (referred here as group 3). Each Berberol K^®^ 1.25 g tablet contained 730 mg of Berberine Phytosome™, and 2.9 mg of total monacolins derived from *Monascus purpureus*. Participants were aged 18–75 years, and supplementary therapy was advised with no restrictions on BMI, alcohol consumption, or smoking habits. However, those on uric acid-lowering drugs and the presence of oncological, neurological, or bowel inflammatory diseases, were not considered for supplementary therapy. At baseline, when supplemental therapy was prescribed, all participants were on a Mediterranean diet and were either statins alone or statins with ezetimibe treatment. Twenty participants had T2DM (diagnosed for at least 8 years) and were on metformin (1,500 mg/day). Participants were advised to maintain their regular diets and treatment during the supplementary therapy.

The study was performed in accordance with the guidelines of Declaration of Helsinki, and approved by the Ethics Committee for Human Experimentation (CESU) of the University of Urbino “Carlo Bo”, Italy. The study was registered in clinicaltrial.gov (identifier NCT06652035).

### 2.4 Outcome measures

The study primary aim across all three cohorts was to assess the effect of daily supplemental QP (containing 400 mg pure quercetin) intake for 90 days on plasma uric acid levels. Secondary outcomes included the potential effects of QP on plasma cholesterol, triglycerides, glycemia, liver enzymes, creatine phosphokinase (CPK), and insulin levels. Compliance, tolerability, and the occurrence of side effects were also evaluated as secondary endpoints.

### 2.5 Statistical analysis

For statistical comparisons between the two treatment groups (Cohort 1 and Cohort 3), and across time points from day 1 to day 90 (Cohort 2), a Split-Plot Design and a mixed analysis of variance (ANOVA) were employed. In cases of significant interaction, Tukey's Multiple HSD Comparison Test was applied. Statistical significance was set at *p* < 0.05. All analyses were performed using JMP 10 statistical software for Mac OS X (LLC 920 SAS Campus Drive, Cary, NC 27513, USA).

## 3 Results

### 3.1 Cohort 1

**Figure 1:**
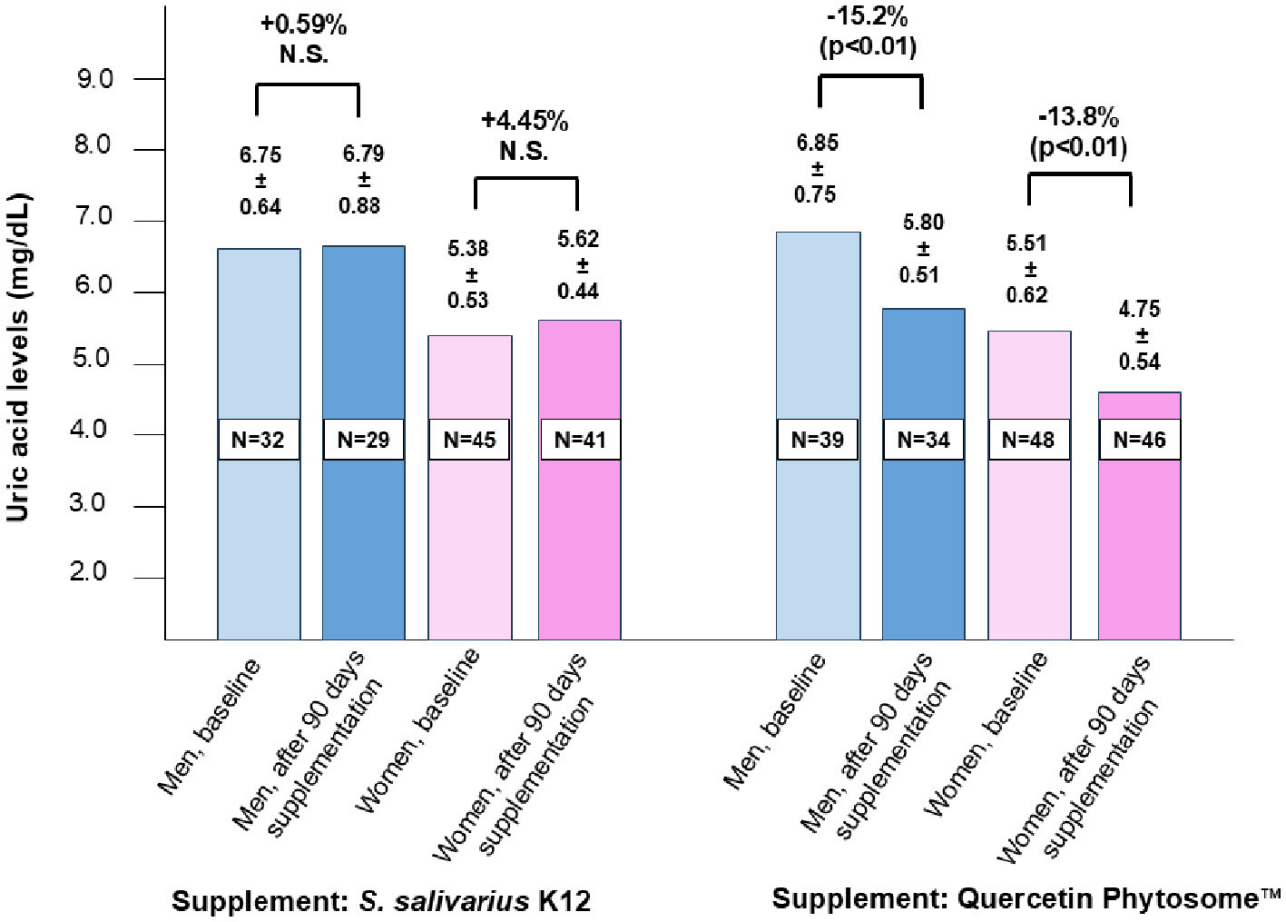
Effect of a 90-day intake of oral supplemental Quercetin Phytosome™ (Quevir^®^ 500 mg, 1 tablet twice daily) (*n* = 87) or probiotic *S. salivarius* K12 (Bactoblis^®^ 1,000 mg, 1 tablet daily) (*n* = 77) on plasma uric acid levels in a cohort of healthy adults. The supplementary therapy was intended to enhance protection against SARS-CoV-2 infection or prevent severe COVID-19 illness. Values above the bars represent median uric acid levels ± standard deviation, while the percentages above the figure denote the relative change between pre- and post-treatment measurements. Statistical significance is also indicated.

At baseline and after 90 days of supplementation, both groups showed no significant differences in age, BMI, cholesterol, triglycerides, glycemia, liver enzymes, CPK, or insulin levels (Table 1). Most participants reported adherence to the supplement regimen. Reported side effects were mild, transient, and comparable across both treatment groups (Supplementary Table S1). As illustrated in Figure 1, both men and women in the QP-treated group exhibited significant reductions in plasma uric acid levels (15.2% in men and 13.8% in women) compared to baseline, while no significant changes were observed in the probiotic K12 group. Although not statistically significant (*p* < 0.062), triglyceride levels were also concurrently reduced in participants treated with QP.

**Table 1:** Effect of oral QP or probiotic *S. salivarius* K12 supplement intake daily for 90 days on metabolic health of healthy adults (*n* = 164).

**Parameter**	**K12-treated**	**K12-treated**	**QP-treated**	**QP-treated**
	**Baseline**	**After 90-days treatment**	**Baseline**	**After 90-days treatment**
Age (years)	56.7 ± 12.5	–	59.5 ± 13.1	–
BMI (Kg/m^2^)	26.2 ± 2.9	25.8 ± 3.2	24.6 ± 3.4	23.9 ± 3.0
Total cholesterol (mg/dL)	187.8 ± 15.5	180.5 ± 16.7	178.6 ± 22.3	184.3 ± 19.8
LDL cholesterol (mg/dL)	95.2 ± 11.6	97.5 ± 8.9	91.8 ± 9.7	93.4 ± 10.8
HDL cholesterol (mg/dL)	42.8 ± 6.7	39.8 ± 7.2	40.5 ± 8.1	43.4 ± 8.8
Triglycerides (mg/dL)	165.4 ± 32.7	169.9 ± 38.2	159.1 ± 35.6	147.3 ± 31.4
Basal glycemia (mg/dL)	89.1 ± 10.6	92.5 ± 8.8	94.6 ± 9.7	97.1 ± 11.4
HbA1c (%)	5.8 ± 0.3	5.7 ± 0.5	5.7 ± 0.6	5.7 ± 0.8
Insulin (μUI/mL)	Not performed	Not performed	Not performed	Not performed
AST (U/L)	23.5 ± 3.5	25.4 ± 3.9	22.8 ± 4.1	21.7 ± 3.8
ALT (U/L)	33.4 ± 4.1	32.8 ± 3.6	34.8 ± 3.9	32.6 ± 3.5
γ-GT (U/L)	42.6 ± 11.8	40.2 ± 16.2	44.1 ± 15.0	42.9 ± 15.2
CPK (U/L)	88.3 ± 14.5	84.0 ± 12.8	92.9 ± 16.7	91.9 ± 14.7

K12, *S. salivarius* K12 (oral colonizing probiotic); QP, Quercetin Phytosome™. BMI, Body Mass Index; LDL, Low Density Lipoprotein; HDL, High Density Lipoprotein; HbA1c, Glycated Hemoglobin; AST, Aspartate aminotransferase; ALT, Alanine aminotransferase; γ-GT, Gamma-Glutamyl Transferase; CPK, Creatine phosphokinase. Values are expressed as average ± standard deviation. The differences observed in the values between the two groups at both times are not significant.

### 3.2 Cohort 2

Table 2 shows that treatment with QP significantly reduced participants serum uric acid levels by 13.1% (*p* < 0.01) and triglyceride levels by 10.2% (*p* < 0.05), while other parameters remained unaffected. Most participants (over 90%) reported adherence to the supplement regimen. The treatment was well-tolerated, with only two participants reporting mild, transient side effects (bloating and heartburn), each lasting no more than 4 days.

**Table 2:** Effect of QP supplementary therapy (daily 400 mg for 90 days) on metabolic health of healthy adults with mildly hyperuricemic (uric acid > 7 mg/dL) with concurrent metabolic disorders (*n* = 22).

**Parameter**	**Baseline**	**After 90-days QP treatment**	***p***
Age (years)	65.6 ± 9.6	–	–
BMI (Kg/m^2^)	Not reported	Not reported	–
Uric acid (mg/dL)	7.45 ± 0.65	6.48 ± 0.70	< 0.01
Total cholesterol (mg/dL)	192.9 ± 42.7	182.8 ± 33.8	n.s.
LDL cholesterol (mg/dL)	112.5 ± 37.4	103.9 ± 32.6	n.s.
HDL cholesterol (mg/dL)	52.7 ± 12.8	53.5 ± 12.8	n.s.
Triglycerides (mg/dL)	143.3 ± 51.1	128.7 ± 30.7	< 0.05
Basal glycemia (mg/dL)	105.5 ± 17.9	101.5 ± 17.9	n.s.
HbA1c (%)	5.9 ± 0.6	5.8 ± 0.7	n.s.
Insulin (μUI/mL)	Not reported	Not reported	–
AST (U/L)	29.0 ± 10.1	29.7 ± 8.4	n.s.
ALT (U/L)	31.2 ± 14.3	31.7 ± 10.8	n.s.
γ-GT (U/L)	45.5 ± 25.8	43.0 ± 15.5	n.s.
CPK (U/L)	88.8 ± 27.8	86.0 ± 29.8	n.s.

QP, Quercetin Phytosome™; BMI, Body Mass Index; LDL, Low Density Lipoprotein; HDL, High Density Lipoprotein; HbA1c, Glycated Hemoglobin; AST, Aspartate aminotransferase; ALT, Alanine aminotransferase; γ-GT, Gamma-Glutamyl Transferase; CPK, Creatine phosphokinase. Values are expressed as average ± standard deviation. n.s., not significant.

### 3.3 Cohort 3

As shown in Table 3, QP treatment (group 1) led to significant reductions in uric acid (−13.7%; *p* < 0.01) and triglyceride levels (−20.8%; *p* < 0.01), with no significant changes observed in other measured parameters. No notable side effects were reported among participants receiving QP. In contrast, participants treated with BM experienced significant reductions in total and LDL cholesterol, as expected, but no changes were observed in uric acid levels for both the hyperuricemic (group 2) and normouricemic (group 3) participants (Table 4). Mild to moderate constipation was reported as a transient side effect by five patients in the hyperuricemic group and seven in the normouricemic group, generally occurring within the 1st week of treatment. This side effect is likely related to berberine's anti-diarrheal properties. No other significant side effects were reported.

**Table 3:** Effect of QP supplementary therapy (daily 400 mg for 90 days) on metabolic health of healthy obese hypercholestrolemic adults with moderate hyperuricemia (uric acid > 7 mg/dL) (*n* = 20).

**Parameter**	**Baseline**	**After 90-days QP treatment**	***p***
Age (years)	51.9 ± 10.2	–	–
BMI (Kg/m^2^)	38.8 ± 6.3	Not reported	–
Uric acid (mg/dL)	8.33 ± 0.64	7.19 ± 0.68	< 0.01
Total cholesterol (mg/dL)	194.3 ± 18.1	186.7 ± 26.9	n.s.
LDL cholesterol (mg/dL)	117.7 ± 20.5	113.2 ± 20.2	n.s.
HDL cholesterol (mg/dL)	46.7 ± 5.9	50.8 ± 7.9	n.s.
Triglycerides (mg/dL)	144.2 ± 54.1	114.2 ± 23.2	< 0.01
Basal glycemia (mg/dL)	96.1 ± 9.7	90.7.5 ± 8.5	n.s.
HbA1c (%)	Not reported	Not reported	-
Insulin (μUI/mL)	16.9 ± 5.4	15.8 ± 5.3	n.s.
AST (U/L)	26.4 ± 5.8	28.4 ± 3.8	n.s.
ALT (U/L)	44.5 ± 12.1	42.6 ± 11.8	n.s.
γ-GT (U/L)	52.7 ± 30.5	52.4 ± 29.6	n.s.
CPK (U/L)	109.5 ± 45.4	118.0 ± 51.7	n.s.

QP, Quercetin Phytosome™; BMI, Body Mass Index; LDL, Low Density Lipoprotein; HDL, High Density Lipoprotein; HbA1c, Glycated Hemoglobin; AST, Aspartate aminotransferase; ALT, Alanine aminotransferase; γ-GT, Gamma-Glutamyl Transferase; CPK, Creatine phosphokinase. Values are expressed as average ± standard deviation. n.s., not significant.

**Table 4:** Effect of 90-day oral supplement therapy of combined 730 mg of berberine Phytosome™ and 2.9 mg of total monacolins (Berberol^®^ K 1.25 g, BM) on metabolic on metabolic health of obese hypercholestrolemic hyperuricemic (*n* = 22) and normouricemic (*n* = 22) healthy adults.

**Parameter**	**Hyperuricemic participants (uric acid** > **8.0 mg/dL)**		**Normouricemic participants (uric acid**<**7.0 mg/dL)**	
	**Baseline**	**After 90 days of BM treatment**	**Baseline**	**After 90 days of BM treatment**
Age (years)	55.9 ± 9.7	–	56.0 ± 10.1	–
BMI (Kg/m^2^)	33.8 ± 5.2	–	34.4 ± 5.2	–
Uric acid (mg/dL)	8.25 ± 0.35	8.23 ± 0.30	5.49 ± 0.95	5.45 ± 1.15
Total cholesterol (mg/dL)	232.3 ± 25.1	202.8 ± 28.8°	233.3 ± 24.3	209.0 ± 25.5^*^
LDL cholesterol (mg/dL)	158.5 ± 22.3	128.4 ± 18.6^*^	159.1 ± 26.8	125.3.5 ± 24.7^*^
HDL cholesterol (mg/dL)	Not performed	Not performed	41.4 ± 17.9	47.8 ± 11.9
Triglycerides (mg/dL)	141.9 ± 40.1	129.3 ± 37.8	133.1 ± 58.9	130.7 ± 55.5
Basal glycemia (mg/dL)	87.7 ± 6.8	83.0 ± 6.9	90.4 ± 10.7	87.2 ± 10.2
HbA1c (%)	Not performed	Not performed	Not performed	Not performed
Insulin (μUI/mL)	Not performed	Not performed	8.2 ± 4.2	8.0 ± 3.4
AST (U/L)	23.9 ± 5.6	21.1 ± 3.0	24.2 ± 8.5	24.7 ± 14.7
ALT (U/L)	24.4 ± 11.0	24.0 ± 8.1	26.4 ± 12.1	26.5 ± 19.1
γ-GT (U/L)	Not performed	Not performed	29.1 ± 21.1	35.2 ± 33.1
CPK (U/L)	124.8 ± 65.3	119.5 ± 50.5	Not performed	Not performed

BMI, Body Mass Index; LDL, Low Density Lipoprotein; HDL, High Density Lipoprotein; HbA1c, Glycated Hemoglobin; AST, Aspartate aminotransferase; ALT, Alanine aminotransferase; γ-GT, Gamma-Glutamyl Transferase; CPK, Creatine phosphokinase. Values are expressed as average ± standard deviation. ^*^p < 0.05; °p < 0.01.

## 4 Discussion

The study based on three distinct cohorts of healthy adults aimed to assess the potential pharmacological effect of QP in controlling hyperuricemia, from real-life settings clinical data. In the first cohort study, a total of 164 healthy adults who received either supplemental QP or probiotic *S. salivarius* K12 in response to the need for added protection against SARS-CoV-2 infection during the COVID-19 pandemic, were studied. These participants had been vaccinated, and none experienced severe COVID-19 outcomes. Analysis of their clinical data revealed that only QP treatment was associated with a statistically significant reduction in serum uric acid levels, with a non-significant trend toward reduced triglycerides. Conversely, K12 administration did not yield any notable metabolic effects.

The second cohort study assessed the hypouricemic effect of QP in 22 healthy adults with mild hyperuricemia and metabolic disorders, all of whom had previously never been treated for their hyperuricemia. In this cohort, QP also demonstrated a significant hypouricemic effect alongside excellent safety and tolerability, comparable to that observed in the first cohort. Furthermore, treatment with QP resulted in a significant reduction in triglycerides, reinforcing the findings from the initial study. This progression from the first to the second cohort highlights QP's consistent efficacy in reducing uric acid and triglyceride levels across different populations, further supporting its potential as a therapeutic agent for hyperuricemia.

The third cohort study evaluated the potential hypouricemic effect of QP in 64 healthy adults with metabolic diseases, including obesity, hypercholesterolemia, and T2DM, and never previously treated for their hyperuricemia. Consistent with the results from the first and second cohorts, participants receiving QP demonstrated a significant reduction in uric acid levels as well as triglycerides, which decreased by 20.8% (*p* < 0.01), together with excellent safety and tolerability. In contrast, no such effect was observed in participants who received the BM supplement. These findings further confirm the hypouricemic potential of quercetin, particularly when delivered in a highly bioavailable form, such as Quercetin Phytosome™. This phospholipid-based carrier system significantly enhances the absorption of quercetin, thereby improving its therapeutic efficacy.

To our knowledge, this is the second study to report the potential hypouricemic pharmacological effect of quercetin, together with favorable safety and tolerability profile, in healthy adults with various metabolic health conditions, based on data collected from real-life clinical settings. The results of the present study align with those of the only previously reported randomized, double-blind, placebo-controlled, crossover trial conducted in the UK (26), further supporting the hypouricemic pharmacological role of QP.

Regarding its mechanism of action, studies suggest that quercetin, and its metabolite quercetin-3′-sulfate, exhibit antihyperuricemic effect through the inhibition of XO (27, 28), rather than by targeting other potential enzymes such as adenosine deaminase (ADA) or purine nucleoside phosphorylase (PNP).

The clinical role of quercetin as a potential safe hypouricemic is significant, particularly for individuals with metabolic diseases who are often prescribed multiple medications. Quercetin presents a safe and effective intervention that may help control uric acid levels without the significant adverse effects associated with traditional pharmacological treatments. By offering a well-tolerated option for managing hyperuricemia, quercetin could help improve the quality of life for patients navigating the complexities of polypharmacy while addressing their metabolic health conditions. This makes QP a valuable addition to therapeutic strategies aimed at mitigating the risks associated with hyperuricemia and its complications.

In addition to the hypouricemic effect of QP, its triglyceride-lowering effect observed in the present study is noteworthy. While participants in Cohort 3 and some in Cohort 2 were on statin therapy, the consistent triglyceride-lowering effect observed across all cohorts, including participants not on statin therapy, supports the independent role of QP in modulating triglyceride levels. The selective reduction in triglyceride levels, with no significant changes in total cholesterol, LDL cholesterol, or HDL cholesterol, suggests that QP may primarily affect triglyceride metabolism. This specificity may be linked to quercetin's direct influence on hepatic triglyceride synthesis and clearance pathways, distinct from the broader lipid-lowering effects of statins. The most pronounced effect of quercetin on triglyceride levels was seen in metabolically compromised obese participants (cohort 3). This finding aligns with previous research conducted in diet-induced obesity models in mice (53). Quercetin consumption resulted in a significant reduction in plasma triglyceride levels by −19% (*p* < 0.05), without affecting food intake, body composition, or energy expenditure. The triglyceride-lowering effect of quercetin could be due to several mechanisms, as below (42–44, 46, 47, 53):

- Downregulation of sterol regulatory element-binding protein-1c (SREBP-1c): Reduces lipogenesis and hepatic triglyceride synthesis.
- Activation of AMPK: Promotes fatty acid oxidation and increases energy expenditure.
- Modulation of adipocyte function: Regulates adipocytokines such as leptin and adiponectin, enhancing lipolysis and reducing fat storage.
- Anti-inflammatory effects: Mediated *via* NF-κB and MAPK, indirectly improving lipid metabolism and contributing to TG reduction.

Epidemiological studies have consistently shown a link between plasma uric acid levels and multiple cardiovascular risk factors, highlighting uric acid as a potential contributor to cardiovascular risk. The recent statement from the Associazione Nazionale Medici Cardiologi Ospedalieri (ANMCO) provides an updated review of evidence supporting the association between elevated uric acid levels and cardiovascular disease risk, as well as the safety and efficacy of uric acid-lowering agents, such as allopurinol and febuxostat, particularly in patients with urate crystal deposits (54). These results highlight QP's potential as a safe and effective triglyceride-lowering agent, which could help mitigate residual cardiovascular risk in individuals with well-controlled cholesterol levels but poor control over triglycerides (55). Furthermore, experimental studies have shown that quercetin can reduce atherosclerosis, supporting its potential role in cardiovascular protection (54).

This study has several limitations that could be addressed in future research, including the absence of randomization, a double-blind, placebo-controlled design, and a relatively small sample size. Furthermore, the lack of a control group treated with a standard hypouricemic drug, such as allopurinol, limited the ability to compare side effects directly. However, despite these limitations, the study's real-life setting and broad inclusion criteria enhance the generalizability of the findings. Future studies incorporating more rigorous trial designs and larger, diverse populations could provide a more comprehensive understanding of QP's hypouricemic and triglyceride-lowering effects.

## 5 Conclusions

The findings from this study suggest that QP exhibits a significant hypouricemic effect in healthy adults with various metabolic health conditions, alongside notable triglyceride-lowering properties. The consistent results across three diverse cohorts indicate QP's potential as a safe, well-tolerated, and effective therapeutic intervention for managing hyperuricemia and triglyceride levels, even in the context of polypharmacy. QP could be considered as a standalone treatment for individuals with mild to moderate hyperuricemia or as an adjunctive therapy for those requiring additional pharmacological interventions. The real-life clinical setting further supports the applicability of QP for broader population use, although future randomized, double-blind, placebo-controlled studies with larger sample sizes and comparison groups are needed to strengthen the evidence on its clinical efficacy and safety profile. QP represents a promising addition to therapeutic strategies aimed at improving metabolic health and cardiovascular outcomes in populations at risk.

## Data Availability

The original contributions presented in the study are included in the article/Supplementary material, further inquiries can be directed to the corresponding author/s.
