# Supplementary material for: Potential pharmacological effect of Quercetin Phytosome™ in the management of hyperuricemia: results from real-life clinical studies
Source: Front Nutr. 2025 Feb 7;12:1519459. doi: 10.3389/fnut.2025.1519459 (PMC11844220; doi:10.3389/fnut.2025.1519459)
Supplement: Supplementary file 1 [file Table_1.docx]

**Supplementary Table 1**. Gut and extra-gut side effects (unexpected and different from those historically reported) recorded during the 90 days supplement intake in the study cohort 1.

Number of individuals are shown.

| **Side-effect** | **K12-treated** | **QP-treated** |
| --- | --- | --- |
| Constipation (C) | 4 | 7 |
| Severity of C* | 3 (I); 1 (II) | 3 (I); 4 (II) |
| Diarrhea (D) | 2 | 2 |
| Severity of D* | 1 (I); 1 (II) | 1 (I); 1 (II) |
| Bloating (B) | 3 | 5 |
| Severity of B* | 3 (I) | 4 (I); 1 (II) |
| Gastric discomfort | 1 | 2 |
| Migraine/headache | 1/1 | 0/2 |
| Mild insomnia | 2 | 2 |
| Skin rash | 0 | 0 |

K12: *S. salivarius* K12 (oral colonizing probiotic); QP: Quercetin Phytosome™.* The numbers from I to IV indicate the severity of the disorder (where I is mild and IV is severe). The differences observed in the values between the two groups are not significant.
